# CD36/Sirtuin 1 Axis Impairment Contributes to Hepatic Steatosis in ACE2-Deficient Mice

**DOI:** 10.1155/2016/6487509

**Published:** 2016-12-22

**Authors:** Valéria Nunes-Souza, Natalia Alenina, Fatimunnisa Qadri, Josef M. Penninger, Robson Augusto S. Santos, Michael Bader, Luiza A. Rabelo

**Affiliations:** ^1^Max Delbrück Center for Molecular Medicine, Berlin, Germany; ^2^Laboratório de Reatividade Cardiovascular (LRC), Núcleo de Síndrome Metabólica, Universidade Federal de Alagoas, Maceió, Brazil; ^3^National Institute of Science and Technology in Nano-Biopharmaceutics (N-BIOFAR), Belo Horizonte, Brazil; ^4^Departamento de Fisiologia e Farmacologia, Centro de Biociências (CB), Universidade Federal de Pernambuco, Recife, Brazil; ^5^Universidade Federal de Minas Gerais, Belo Horizonte, Brazil; ^6^Institute of Molecular Biotechnology of the Austrian Academy of Sciences, Vienna, Austria; ^7^Charité–University Medicine Berlin, Berlin, Germany; ^8^Institute for Biology, University of Lübeck, Lübeck, Germany; ^9^German Center for Cardiovascular Research (DZHK), Berlin, Germany

## Abstract

*Background and Aims*. Angiotensin converting enzyme 2 (ACE2) is an important component of the renin-angiotensin system. Since angiotensin peptides have been shown to be involved in hepatic steatosis, we aimed to evaluate the hepatic lipid profile in ACE2-deficient (ACE2^−/y^) mice.* Methods*. Male C57BL/6 and ACE2^−/y^ mice were analyzed at the age of 3 and 6 months for alterations in the lipid profiles of plasma, faeces, and liver and for hepatic steatosis.* Results*. ACE2^−/y^ mice showed lower body weight and white adipose tissue at all ages investigated. Moreover, these mice had lower levels of cholesterol, triglycerides, and nonesterified fatty acids in plasma. Strikingly, ACE2^−/y^ mice showed high deposition of lipids in the liver. Expression of CD36, a protein involved in the uptake of triglycerides in liver, was increased in ACE2^−/y^ mice. Concurrently, these mice exhibited an increase in hepatic oxidative stress, evidenced by increased lipid peroxidation and expression of uncoupling protein 2, and downregulation of sirtuin 1. ACE2^−/y^ mice also showed impairments in glucose metabolism and insulin signaling in the liver.* Conclusions*. Deletion of ACE2 causes CD36/sirtuin 1 axis impairment and thereby interferes with lipid homeostasis, leading to lipodystrophy and steatosis.

## 1. Introduction

Nonalcoholic fatty liver disease (NAFLD), a metabolic disorder of increasing clinical importance with different pathological presentations varying from initial hepatic steatosis, through nonalcoholic steatohepatitis, to fibrosis and cirrhosis, has been considered a novel component of the metabolic syndrome (MetS) [1, 2]. MetS is characterized by a cluster of cardiovascular and metabolic disorders, including central obesity, insulin resistance, glucose intolerance, dyslipidemia, and hypertension [1, 2]. Emerging evidence indicates that the renin-angiotensin system (RAS) plays an important role in the pathogenesis of MetS and NAFLD [3–6]. The “classical arm” (angiotensin converting enzyme/angiotensin II/AT1 receptor [ACE/AngII/AT1]) promotes the disease [4–6], whereas the “protective arm” (angiotensin converting enzyme 2/angiotensin-(1–7)/Mas receptor [ACE2/Ang-(1–7)/Mas]) counteracts it [3, 7, 8].

ACE acts on angiotensin I to form the AngII, a molecule which constricts vessels after binding to the AT1 receptor in arterioles [9, 10]. Beyond that, AngII has other functions in the cardiovascular system that promote elevated blood pressure, such as increased release of aldosterone and vasopressin, which increase sodium and water reabsorption, respectively, in the renal distal tubules [11]. Therefore, ACE2 is an important enzyme that negatively regulates the RAS, through reduction of AngII and increase of Ang-(1–7), a vasodilator molecule, conferring ACE2 a protective role in cardiovascular diseases [10, 11].

Metabolic studies demonstrated an important role of the ACE2/Ang-(1–7)/Mas pathway in the maintenance of homeostasis [3, 8, 12, 13]. Mice deficient for Mas presented dyslipidemia and hyperglycemia [12]. Moreover, rats overexpressing Ang-(1–7) showed an improvement in glucose tolerance and insulin sensitivity and also exhibited decreased triglycerides, cholesterol, and abdominal fat mass [8]. Treatment of diabetic rats with an oral formulation of Ang-(1–7) resulted in drastic reductions in glycemia and an increase in insulin sensitivity, also implying insulin resistance under high fat conditions [14]. Interestingly, ACE2 is not only a protease which metabolizes peptides, such as AngII, apelin, and des-Arg^9^-bradykinin [15], but is also involved in the resorption of large amino acids from the gut [16, 17]. In this regard, ACE2 deletion leads to defects in amino acid uptake and intestinal inflammation, but effects on lipid metabolism have not yet been reported.

AngII has been shown to cause NAFLD [18], whereas Ang-(1–7) elicits opposite effects [3]. Accordingly, both ACE inhibitors and AT1 antagonists protect from fatty liver and fibrosis [18], recombinant ACE2 has beneficial effects on hepatic fibrosis in mice [19], and during the preparation of this manuscript, Cao and collaborators [20] showed that ACE2/Ang-(1–7)/Mas axis may reduce liver lipid accumulation. On the other hand, the underlying mechanisms are not yet well understood but, of the many factors that stimulate this process, redox balance seems to be one of the most important in the liver [21, 22].

Reactive oxygen species (ROS), such as nitric oxide (^∙^NO) superoxide anion (^∙^O_2_^−^) and hydrogen peroxide (H_2_O_2_), are crucial mediators of angiotensin peptide actions [10], since AngII promotes their generation [23] and Ang-(1–7) reduces oxidative stress [10]. ROS are chronically elevated in NAFLD and contribute to the pathogenesis of the disease [21, 22]. However, it is still unknown whether ACE2 plays a role in this liver disorder or whether ACE2 deletion interferes with regulation of key factors of lipid metabolism, such as fatty acid translocase, also called cluster of differentiation 36 (CD36), peroxisome proliferator-activated receptor *γ* (PPAR*γ*), adipocyte protein 2 (aP2), fatty acid synthase (FAS), and sirtuin 1, as well as of key moderators of ROS production in hepatic metabolism, such as uncoupling protein type 2 (UCP2). Taking in consideration the important role of angiotensins and ROS in the development of NAFLD [21, 22], we aimed to investigate the hepatic lipid profile in ACE2-deficient mice in which the relative abundance of AngII and Ang-(1–7) is distorted. Indeed, we found that the deletion of ACE2 causes hepatic steatosis accompanied by an impairment of the CD36/sirtuin 1 axis, insulin signaling, and glucose metabolism in the liver. These results reveal a central role of ACE2 in lipid homeostasis, preventing lipodystrophy probably by decreasing the levels of AngII and/or increasing Ang-(1–7) in the liver.

## 2. Methods

### 2.1. Animals and Experimental Procedures

C57BL/6 (WT) and ACE2-deficient (ACE2^−/y^) male mice on C57BL/6 genetic background [9] at 3 and 6 months of age were used in this study under standard diet (10% kcal from fat) from SSNIFF® (Soest, Germany). The mice were kept in a 12-hour light/dark cycle, with controlled humidity and temperature environment and fed* ad libitum.* All experiments were approved by the “Landesamt für Gesundheit und Soziales” (LAGeSo; Berlin) and performed in accordance with the “Guide for the Care and Use of Laboratory Animals” (NIH publication 86–23, 1996).

### 2.2. Euthanasia and Organ Collection

After intraperitoneal (i.p) anesthesia using a xylazine/ketamine solution (10/110, mg·kg^−1^), 12 h-fasted animals were euthanized by exsanguination through cardiac puncture of the right ventricle. The whole blood was collected and centrifuged (4,000 rpm for 10 min), and the plasma was separated and stored at −80°C. The animals were perfused with heparinized saline and, in sequence, the liver and white adipose tissue (WAT) were carefully removed, weighed, immediately frozen in dry ice and stored at −80°C until quantitative PCR, western blot and the other analyses were performed. WAT index was calculated using the following formula: WAT index (%) = {(epididymal fat + perirenal fat)/(body weight)}*∗*100.

### 2.3. Biochemical Analyses

Nonesterified fatty acid (NEFA) kit was used to measure plasma and liver NEFA concentrations (Wako Chemicals GmbH®, Neuss, Germany). The total cholesterol (TCOL) and triglycerides (TG) levels were assayed with commercials kits (Labtest®, Belo Horizonte, Brazil), following the manufacturers' instructions with adaptations for microplates. All measurements were performed with a TECAN® Infinite 200 PRO plate reader (Männedorf, Switzerland).

### 2.4. Evaluation of Liver Injury

Liver injury (the degree of hepatocellular damage) was assessed by measuring the enzymatic activities of alanine aminotransferase (ALT) and aspartate aminotransferase (AST) in plasma with commercial kits (Labtest, Belo Horizonte, Brazil).

### 2.5. Liver and Fecal Lipids Analysis

The total hepatic and fecal lipids were extracted according to a gravimetric standard method [24]. Total lipids were measured by weighing the samples on an analytical balance after extraction, being normalized by the mass of faeces used for extraction. After this, the total lipids were diluted in isopropanol and measured by commercial kits for TCOL, TG (Labtest, Belo Horizonte, Brazil), and NEFA (Wako Chemicals GmbH, Neuss, Germany).

### 2.6. Liver and WAT Histological Analysis

Fragments of liver and WAT were fixed in 4% buffered formaldehyde, embedded in paraffin and sectioned at 3 *μ*m and 10 *μ*m, respectively. WAT was stained with H&E in order to determine adipocyte diameters. The lipid deposition in the liver was analyzed indirectly by immunofluorescence staining for adipophilin. In brief, sections were deparaffinized, rehydrated, and boiled in citrate buffer, pH 7, for 20 min in a vegetable steamer. The sections were incubated with an antibody against adipophilin (1 : 500, Fitzgerald, Acton, USA) overnight at 4°C. The sections were then incubated with a secondary antibody conjugated with Cy3 (1 : 300) and coverslipped using the mounting medium “Vectashild with DAPI-Hard set” (Vector Lab). The sections were observed under a Keyence® microscope (BZ 9000, Osaka, Japan). Digital photographs were taken from each section; adipocyte diameter and adipophilin expression were quantified using the “BZ II Analyzer” image processing software (Keyence BZ 9000 Software, Osaka, Japan).

### 2.7. Lipid Peroxidation, Superoxide Dismutase, and Catalase Activity Measurement in the Liver

The hepatic lipid peroxidation was quantified by measuring the ThioBarbituric Acid-Reactive Substances (TBARS), a marker of oxidative stress which was assayed by malondialdehyde (MDA) in liver homogenates as described [25]. Total superoxide dismutase (SOD) activity was assessed with a commercial colorimetric kit (Sigma-Aldrich, Seelze, Germany). Catalase activity was measured according to Xu et al. [26]. All results were normalized to protein concentration [27].

### 2.8. Gluconeogenesis

Gluconeogenesis was evaluated by the pyruvate test. It was performed after 16 hours overnight fast. The animals were weighed and blood was collected from the tail vein to measure glucose before the injection of 2 g sodium pyruvate·kg^−1^ (i.p) (Sodium Pyruvate, Sigma-Aldrich, Seelze, Germany). After injection, the glucose levels were measured at 15, 30, 60, 90, and 120 minutes.

### 2.9. Real Time Quantitative PCR

Total RNA was isolated from liver using Trizol (TRizol® Reagent, Invitrogen, Darmstadt, Germany) and subsequently cleaned using RNeasy Mini kit (Qiagen, Hilden, Germany). RNA concentration was quantified using spectrophotometry (NanoDrop®, München, Germany) and 1 *μ*g was taken for the synthesis of cDNA using M-MLV Reverse Transcriptase (Invitrogen). The reaction product was amplified using the GoTaq qPCR Master Mix (Promega®; Mannheim, Germany) by real time quantitative PCR (ABI 7900HT Real-Time PCR System-Applied Biosystems, Darmstadt, Germany) with gene-specific primers (sequences listed in Table  1; Supplemental Data in Supplementary Material available online at http://dx.doi.org/10.1155/2016/6487509). The mRNA expression level was quantified by normalization to the internal reference, GAPDH, using the 2^−ΔΔCt^ method [28].

### 2.10. Western Blotting

For Western blotting, proteins were isolated using a lysis buffer (Cell Signaling Technology®, Beverly, MA) containing mammalian protease and phosphatase inhibitor cocktail (Roche®, Mannheim, Germany) and quantified by Bradford assay [27]. The proteins were separated by electrophoresis, transferred to a polyvinylidene fluoride membrane, which was blocked by incubation in Odyssey® blocking buffer (Li-COR, Biosciences, Lincoln, USA) for 1 h at room temperature (RT). Thereafter, the membrane was probed (overnight, 4°C) with one of the following primary antibodies: UCP2 (1 : 500), sirtuin 1 (1 : 1,000), *α*-IRS-1 (1 : 1,000), PI3-K (1 : 500), AKT (1 : 1,000), phospho-GSK 3*β* (1 : 1,000), and GSK 3*β* (1 : 1,000) followed by incubation with a secondary antibody for 1 h at RT. Band intensities were acquired and quantified using the Odyssey infrared imaging system (Li-COR, Biosciences, Lincoln, USA). The membrane was stripped and reprobed with *β*-actin (1 : 1,000) antibody to obtain an endogenous control for protein quantification.

### 2.11. Statistical Analysis

Data are expressed as mean ± standard error of the mean (SEM). Student's* t*-test was performed for the between-group comparisons (Graph Pad Prism® 5.0, San Diego, CA, USA). The hepatic gluconeogenesis test was analyzed by two-way ANOVA followed by* Bonferroni's *post-test. *p* < 0.05 was considered statistically significant.

## 3. Results

### 3.1. ACE2 Deficiency Decreases Body Weight and Changes the Plasma Lipid Profile

**Figure 1:**
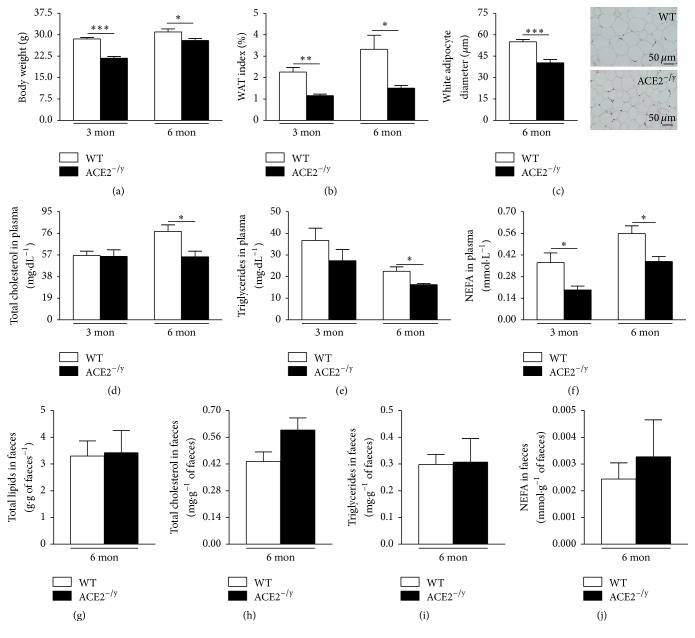
Body weight, WAT index and lipid profile in ACE2^−/y^ mice. (a) Body weight (g); (b) WAT index (%) of WT and ACE2^−/y^ mice at the age of 3 and 6 months; (c) white adipocyte diameter (*μ*m); (d) total cholesterol (mg·dL^−1^), (e) triglycerides, (mg·dL^−1^), (f) nonesterified fatty acids (NEFA) (mmol·L^−1^) in plasma of WT and ACE2^^−/y^^ mice at the age of 3 and 6 months; (g) total lipids (g·g^−1^ of faeces), (h) total cholesterol (mg·g^−1^ of faeces), (i) triglycerides (mg·g of faeces^−1^), and (j) NEFA (mmol·g of faeces^−1^) in faeces of WT and ACE2^−/y^ mice at the age of 6 months. Each bar graph represents the mean ± SEM. Student's* t* test: ^*∗*^ *p* < 0.05; ^*∗∗*^ *p* < 0.01; ^*∗∗∗*^ *p* < 0.001.

To reveal the role of ACE2 in fat metabolism, we evaluated body (BW) and white adipose tissue (WAT) weight and plasma lipid profile in ACE2^−/y^ mice. These animals showed lower BW and WAT index at 3 and 6 months of age compared to WT mice (Figures 1(a) and 1(b)) and a decrease in white adipocyte diameter (Figure 1(c)). The reduction in these parameters was accompanied by decreased lipids in plasma. In 3- and 6 month-old mice, the levels of NEFA in plasma were significantly lower, and at 6 months of age also TCOL and TG were reduced in ACE2^−/y^ mice compared to WT animals (Figures 1(d)–1(f)).

### 3.2. ACE2 Deficiency Leads to Hepatic Steatosis and Oxidative Stress

**Figure 2:**
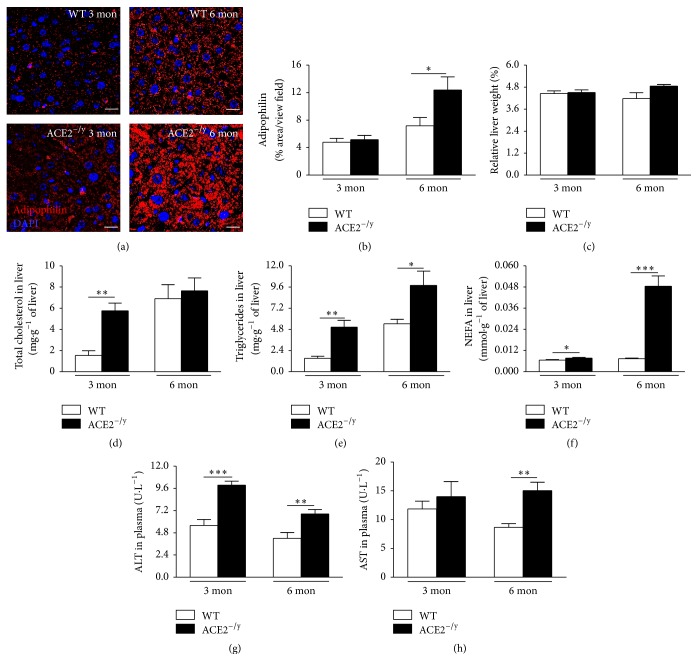
Hepatic steatosis and liver function of ACE2^−/y^ mice. (a) Immunofluorescence staining for adipophilin in the liver; (b) adipophilin quantification (% area/view field); (c) relative liver weight (%); (d) total cholesterol in liver (mg·g^−1^ of liver); (e) triglycerides in liver (mg·g^−1^ of liver); (f) nonesterified fatty acids (NEFA) in liver (mmol·g^−1^ of liver); (g) alanine aminotransferase (ALT) in plasma (U·L^−1^); (h) aspartate aminotransferase (AST) in plasma (U·L^−1^) of WT and ACE2^−/y^ mice at the age of 3 and 6 months. Each bar graph represents the mean ± SEM. Student's* t* test: ^*∗*^ *p* < 0.05;^*∗∗*^ *p* < 0.01; ^*∗∗∗*^ *p* < 0.001.

As ACE2^^−/y^^ mice develop intestinal dysfunction [16, 17], we investigated whether the missing plasma lipids were released to the faeces in 6-month-old mice. The results showed that there were no significant differences in total lipids, TCOL, TG, and NEFA levels between WT and ACE2^−/y^ mice (Figures 1(g)–1(j)). However, when we investigated ectopic fat deposition, we identified lipid accumulation in the liver. Immunofluorescence staining for adipophilin, a lipid droplet-associated protein, showed a higher fat deposition in ACE2^−/y^ mice at 6 months of age compared to WT (Figures 2(a) and 2(b)). These data indicate that ACE2^−/y^ mice present a steatotic state.

Although these animals showed no difference in relative liver weight (Figure 2(c)), they stored increased levels of TCOL, TG, and NEFA in the liver at the age of 3 months and of TG and NEFA at the age of 6 months compared to WT (Figures 2(d)–2(f)). Plasma ALT was significantly increased in ACE2^−/y^ mice at both ages (Figure 2(g)), and plasma AST was also significantly increased in 6-month-old ACE2^−/y^ mice (Figure 2(h)), confirming liver injury in these animals.

**Figure 3:**
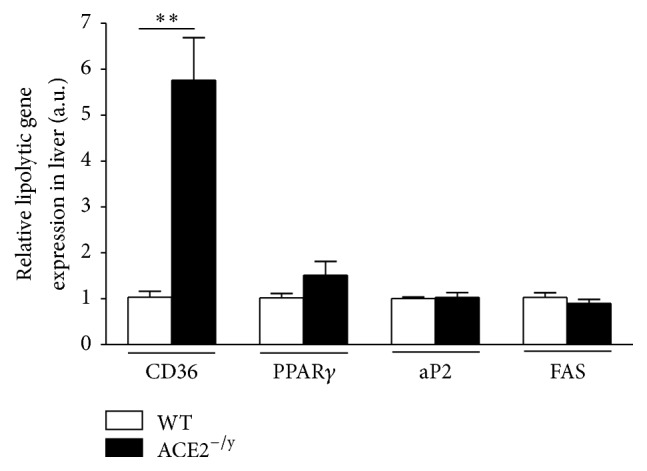
Relative expression of genes involved in the lipolytic pathway in the liver. mRNA expression levels (arbitrary units) of fatty acid translocase (CD36), peroxisome proliferator-activated receptor gamma (PPAR*γ*), adipocyte protein 2 (aP2), and fatty acid synthase (FAS) of WT and ACE2^−/y^ mice at the age of 6 months. Each bar graph represents the mean ± SEM. Student's* t* test: ^*∗∗*^ *p* < 0.01.

Expression analysis of genes, involved in lipid metabolism in the liver, showed that ACE2^−/y^ mice have significantly more mRNA for CD36, but the levels of mRNA for PPAR*γ*, aP2, and FAS in the liver were not different between ACE2^−/y^ and WT mice (Figure 3).

**Figure 4:**
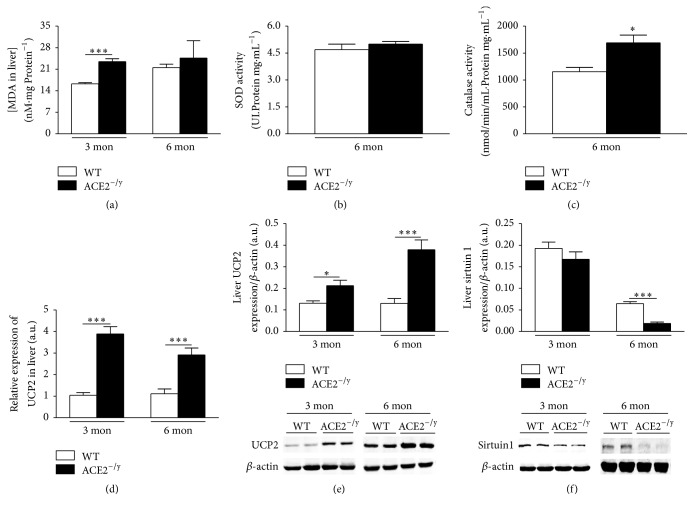
Markers of redox status, antioxidant enzymes, and sirtuin 1 protein expression in liver. (a) Malondialdehyde (MDA) in liver of WT and ACE2^^−/y^^ mice at the age of 3 and 6 months (nM·mg protein^−1^); (b) superoxide dismutase (SOD) activity in the liver (UI·Protein mg·mL^−1^); (c) catalase activity in the liver (nmol/min/mL·Protein mg·mL^−1^) of WT and ACE2^−/y^ mice at the age of 6 months; (d) relative mRNA and (e) protein expression of uncoupling protein 2 (UCP2) in the liver (arbitrary units); (f) sirtuin 1 protein expression in the liver (arbitrary units) of WT and ACE2^−/y^ mice at the age of 3 and 6 months. Each bar graph represents the mean ± SEM. Student's* t* test: ^*∗*^ *p* < 0.05; ^*∗∗∗*^ *p* < 0.001.

ACE2^−/y^ mice showed increased hepatic lipid peroxidation at the age of 3, but not 6 months (Figure 4(a)). Next, we analyzed antioxidant enzymes in liver homogenates. SOD activity showed no difference between ACE2^−/y^ and WT at the age of 6 months. However, catalase activity was significantly higher in ACE2^−/y^ mice compared to WT (Figures 4(b) and 4(c)). In addition, the expression of the UCP2 was significantly higher in ACE2^−/y^ mice compared to WT at mRNA (Figure 4(d)) and protein levels at both ages (Figure 4(e)), suggesting that the steatosis is accompanied by oxidative stress. The levels of sirtuin 1 were significantly decreased in liver of 6- but not 3-month-old ACE2^−/y^ mice compared to WT (Figure 4(f)).

### 3.3. ACE2 Deficiency Leads to Impaired Insulin Signaling in the Liver

**Figure 5:**
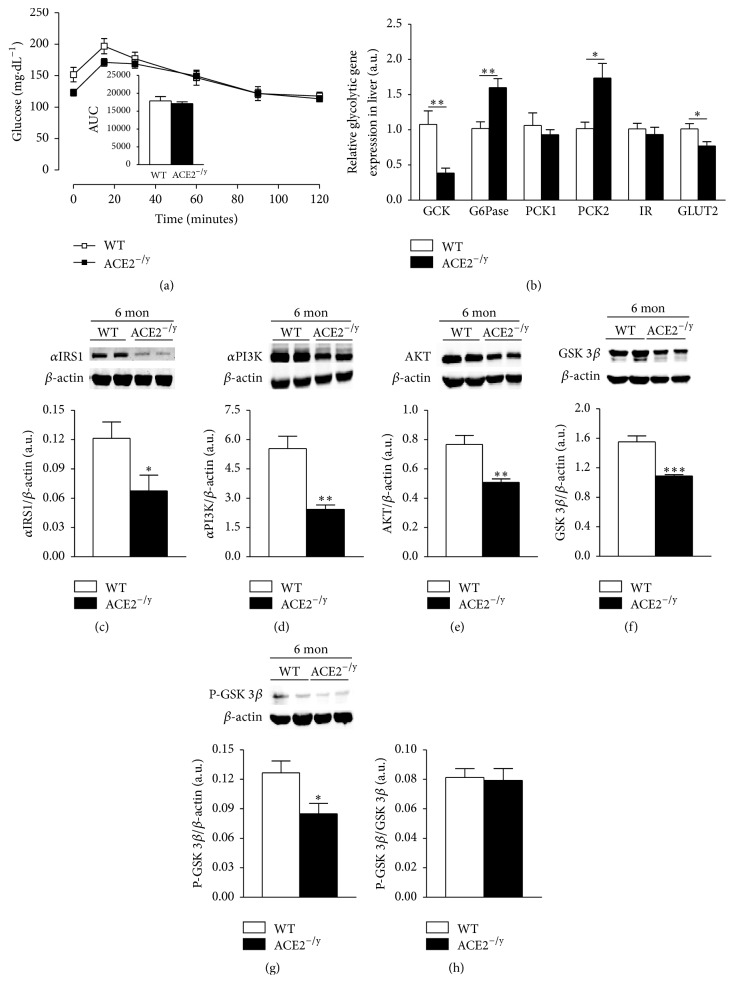
Hepatic glucose metabolism. (a) Evaluation of hepatic gluconeogenesis stimulated by intraperitoneal injection of pyruvate and area under the curve of the test (AUC); (b) relative mRNA expression of glycolytic genes in the liver (glucokinase, GCK; glucose 6-phosphatase, G6Pase; phosphoenolpyruvate carboxykinase 1 and 2, PCK; insulin receptor, IR; and glucose transporter type 2, GLUT2) (arbitrary units); (c) *α*-insulin receptor substrate-1 (*α*IRS) protein expression in the liver (arbitrary units); (d) phosphatidyl inositol-3 kinase (PI3-K) protein expression in the liver (arbitrary units); (e) AKT protein expression in the liver (arbitrary units); (f) glycogen synthase kinase (GSK) 3*β* in the liver (arbitrary units); (g) phospho-GSK 3*β* in the liver (arbitrary units); (h) GSK 3*β*/phospho-GSK 3*β* in the liver (arbitrary units) of WT and ACE2^−/y^ mice at the age of 6 months. Data are presented as mean ± SEM. Student's *t* test: ^*∗*^ *p* < 0.05; ^*∗∗*^ *p* < 0.01; ^*∗∗∗*^ *p* < 0.001.

As steatosis is often associated with insulin resistance [2], we investigated glucose metabolism and insulin signaling in the liver of ACE2^−/y^ mice. In this organ, ACE2^−/y^ mice showed severe impairment in insulin signaling and glucose handling. Whereas the hepatic capacity of glucose production from pyruvate was not altered in these mice (Figure 5(a)), several genes involved in glucose metabolism were dysregulated. ACE2^−/y^ mice presented a reduction in the relative expression of glucokinase (GCK) and of glucose transporter type 2 (GLUT2) and increased levels of expression of glucose 6-phosphatase (G6Pase) and phosphoenolpyruvate carboxykinase subtype 2 (PCK2). The other subtype, PCK1, and the insulin receptor were however not differentially expressed between the groups (Figure 5(b)). Moreover, ACE2^−/y^ mice presented a significant decrease in proteins involved in glycolysis, such as insulin receptor substrate-1 (*α*IRS-1), phosphatidyl inositol-3 kinase (PI3-K), and AKT compared to WT (Figures 5(c)–5(e)). Furthermore, GSK 3*β* and phosphorylated GSK 3*β* were decreased in ACE2^−/y^ mice (Figures 5(f) and 5(g)). However, GSK 3*β*/phosphorylated GSK 3*β* ratio was not different between groups (Figure 5(h)).

## 4. Discussion

The major findings of the present study are that the deletion of ACE2 causes paradoxical metabolic effects: on the one hand, it results in a markedly diminished BW and WAT. On the other hand, it leads to the development of steatosis and insulin resistance in the liver. Evidence for this disorder includes an increased amount of adipophilin-containing vesicles in hepatocytes (Figures 2(a) and 2(b)), augmented lipids in the liver (Figures 2(d)–2(f)), and an accumulation of liver enzymes in plasma as indication of liver injury (Figures 2(g) and 2(h)). The decrease of WAT index, adipocyte diameter (Figures 1(b) and 1(c)), and plasma lipids (Figures 1(d)–1(f)) associated with the normal faecal lipid excretion (Figures 1(g)–1(j)) suggested an increased uptake of fatty acids by the liver as primary cause for the NAFLD observed in ACE2^−/y^ mice. Indeed, CD36, the fatty acid translocase, is upregulated in mice lacking ACE2 (Figure 3). It has been shown that the upregulation of CD36 in the liver is associated with increased steatosis in NAFLD patients [29, 30] and CD36^−/−^ mice are resistant to alcohol and high carbohydrate-induced hepatic steatosis [31]. Moreover, in mice and humans aging increases CD36 membrane expression in the liver [29], causing increased fat uptake and advancing NAFLD with age. The increase in CD36 may be caused by the decreased expression of sirtuin 1 in ACE2^−/y^ mice (Figure 4(f)), as it was observed in heterozygous sirtuin 1 deficient animals [32]. In addition, Cao and collaborators [33] showed that the deletion of hepatocyte-specific menin causes steatosis in aging mice by decreasing the levels of sirtuin 1 in the liver and upregulation of CD36, which demonstrates a metabolic link between CD36 and sirtuin 1. AngII has been shown to downregulate sirtuin 1 in other cell types [34] and Ang-(1–7) exhibit the opposite effect in liver cells [35]. Thus, a downregulation of this translocase can be expected from the imbalance between the two peptides in ACE2^−/y^ mice. Interestingly, it has recently been shown that sirtuin 1 can* vice versa* regulate ACE2 expression [36].

Increase in cytosolic fatty acids leads to mitochondrial damage and the production of reactive oxygen species (ROS) [21]. Moreover, hepatic sirtuin 1 deficiency in mice induces oxidative liver damage [37]. Indeed, we observed increased lipid peroxidation and UCP2 expression as oxidative markers also in the liver of ACE2^−/y^ mice suggesting that steatosis is accompanied by elevated oxidative stress in these animals. The high lipid peroxidation observed in the ACE2^−/y^ mice at the age of 3 months is probably due to a high production of H_2_O_2_ in these mice [38]. This could explain the high catalase activity, which degrades H_2_O_2,_ in an attempt to combat the elevation of this ROS [38]. UCP2, a mitochondrial anion carrier protein [39], plays a key role as a moderator of ROS production in hepatic metabolism [40, 41]. Accordingly, UCP2^−/−^ mice showed increased ROS formation [42]. In ACE2^−/y^ mice, an increase in intracellular lipids in the liver may lead to a mitochondria overload, followed by an increase in ROS production during the *β*-oxidation of lipids, which in turn stimulates the expression of UCP2 to combat this imbalance. These data suggest that the increased expression of this uncoupling protein could be an insufficient defense mechanism in the attempt to prevent the progression of steatosis in ACE2^−/y^ mice [43]. We cannot exclude that AngII-induced oxidative stress may be a primary cause of liver steatosis in ACE2^−/y^ mice, which is not compensated by Ang-(1–7) in these animals. Experimental evidence indicates that RAS signaling plays a critical role in the metabolism of fat in the liver [3, 4, 7, 18, 19, 44]. Moreover, Cao and collaborators [20] recently confirmed that ACE2^−/y^ mice present hepatic steatosis, oxidative stress, and inflammation. This report also showed that Ang-(1–7) and ACE2 ameliorated all of these parameters in a liver cell line. The authors attribute the reduction of liver lipid accumulation, induced by ACE2/Ang-(1–7)/Mas axis, partly to the regulation of lipid-metabolizing genes.

As already described in other mouse models and patients [45], the high lipid deposition in the liver of ACE2^−/y^ mice resulted in impaired insulin signaling and glucose metabolism. Although these animals showed normal glucose production from pyruvate, changes in the expression of important genes for glucose metabolism, such as GCK, G6Pase, PCK2, and GLUT2, suggest that as a result of steatosis, glycolysis could be impaired. Furthermore, the decrease in IRS-1, PI3-K, AKT, and GSK3*β* pathway confirms that insulin signaling is impaired in the liver of ACE2^−/y^ mice. Accordingly, Cao and collaborators [46] showed that the activation of the ACE2/Ang-(1–7)/Mas axis has a beneficial effect on insulin resistance in the liver through reduced oxidative stress in hepatic cells and modulation of the Akt/PI3K/IRS-1/JNK insulin signaling pathway.

ACE-deficient mice show a pronounced increase in expression of key genes involved in lipolysis and fatty acid oxidation in the liver, such as lipoprotein lipase, carnitine palmitoyl transferase, and long-chain acetyl CoA dehydrogenase. This suggests an increase in fatty acid hydrolysis and *β*-oxidation, which could prevent an accumulation of lipids in the liver and might be due to the absence of AngII in these knockout animals [6]. On the other hand, ACE2-deficient mice have increased levels of AngII which is known to contribute to the development of steatosis and insulin resistance [45]. AT1 receptor activation leads to steatosis via decreased UCP2 in a rat model with metabolic syndrome [47], and the deletion of AT1 receptor reduces hepatic steatosis [44]. Moreover, it has been shown that the oral treatment with Ang-(1–7) prevents HFD-induced steatosis [7] and that the deletion of Mas in ApoE-deficient mice leads to an increased hepatic lipid content [3]. Taken together, this large body of experimental evidence and our results show that a balanced activity of the two axes of the RAS, ACE/AngII/AT1 and ACE2/Ang-(1–7)/Mas, is essential to metabolize fat for energy maintenance in the liver without inducing steatosis. The observed BW reduction in ACE2-deficient mice confirms the findings of Singer et al. [17], who link it to the defective amino acid absorption in the gut of these animals. However, the reduction in WAT by the mechanism described in our study may also contribute to this phenotype. Future studies have to validate the proposed mechanisms by which angiotensin peptides regulate lipid metabolism and hepatic oxidative stress.

**Figure 6:**
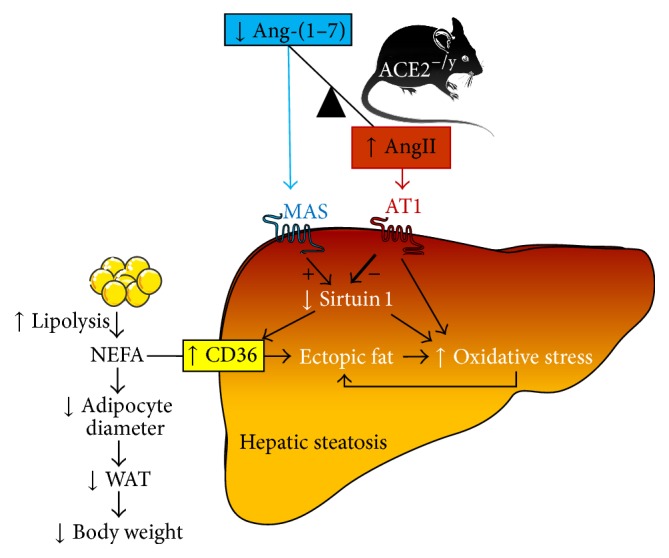
Proposed mechanism of hepatic steatosis in ACE2^−/y^ mice. ACE2 deletion causes an imbalance between Ang-(1–7) and AngII with higher levels of the latter. Since Ang-(1–7) via Mas stimulates and AngII via the AT1 receptor inhibits sirtuin 1 expression, this regulatory factor is downregulated. This leads to upregulation of the fatty acid translocase, CD36, and an increased uptake of nonesterified fatty acids (NEFA) into hepatocytes causing ectopic fat deposition and oxidative stress in the liver. Oxidative stress is augmented by direct effects of the decreased sirtuin 1 and the increased AT1-signalling on the formation of reactive oxygen species. In the blood, NEFAs are decreased leading to shrinking adipocytes and fat pads, as well as a drop in body weight.

In sum, ACE2 deletion causes CD36/sirtuin 1 axis impairment and thereby contributes to the fat deposition in the liver leading to NAFLD, oxidative stress, and impaired insulin signaling (summarized in Figure 6). Therefore, ACE2-deficient mice provide a suitable model for assessing the pathophysiological relevance of NAFLD and represent an excellent tool to investigate new therapeutic strategies for MetS as well as associating disorders.

## Supplementary Material

The primer sequences (Forward and Reverse) used for real time quantitative PCR are described in Table 1.

## Abbreviations

ACE: Angiotensin converting enzyme
ACE2: Angiotensin converting enzyme 2
ALT: Alanine aminotransferase
Ang-(1–7): Angiotensin-(1–7)
AngII: Angiotensin II
aP2: Adipocyte protein 2 (fatty acid binding protein)
AST: Aspartate aminotransferase
AT1: AT1 receptor
BW: Body weight
CD36: Cluster of differentiation 36 (fatty acid translocase)
FAS: Fatty acid synthase
G6Pase: Glucose 6-phosphatase
GAPDH: Glyceraldehyde 3-phosphate dehydrogenase
GCK: Glucokinase
GLUT2: Glucose transporter type 2
GSK: Glycogen synthase kinase
H_2_O_2_: Hydrogen peroxide
H&E: Hematoxylin and eosin
HFD: High fat diet
IR: Insulin receptor
IRS: Insulin receptor substrate
MDA: Malondialdehyde
MetS: Metabolic syndrome
NAFLD: Nonalcoholic fatty liver disease
NEFA: Nonesterified fatty acids
PCK1: Phosphoenolpyruvate carboxykinase 1
PCK2: Phosphoenolpyruvate carboxykinase 2
PI3-K: Phosphatidyl inositol-3 kinase
PPAR*γ*: Peroxisome proliferator-activated receptor *γ*
RAS: Renin-angiotensin system
ROS: Reactive oxygen species
SOD: Superoxide dismutase
TBARS: ThioBarbituric Acid-Reactive Substances
TCOL: Total cholesterol
TG: Triglycerides
UCP2: Uncoupling protein 2
WAT: White adipose tissue.
