# Supplementary material for: CD36/Sirtuin 1 Axis Impairment Contributes to Hepatic Steatosis in ACE2-Deficient Mice
Source: Oxid Med Cell Longev. 2016 Dec 22;2016:6487509. doi: 10.1155/2016/6487509 (PMC5215286; doi:10.1155/2016/6487509)
Supplement: Supplementary file 1 — The primer sequences (Forward and Reverse) used for real time quantitative PCR are described in Table 1. [file 6487509.f1.pdf]

CD36/sirtuin 1 axis impairment contributes to hepatic steatosis in ACE2-deficient mice

Valéria Nunes-Souza[1,2,3], Natalia Alenina[1,3,4*], Fatimunnisa Qadri[1], Josef M Penninger[5], Robson Augusto S Santos[3,4], Michael Bader[1,3,4,6,7], Luiza A Rabelo[1,2,3*]

Supplemental data

Table 1. Primer sequences used for real time quantitative PCR.

Primers	Sequence Forward and Reverse (5' – 3')
aP2	**F:** GTTGAGGATGGTGTCCACGTAC<br>**R:** GTATGCCATGTGTGACCGGAG
CD36	**F:** CATGTAGGAAATGTGGAAGCGA<br>**R:** TCTTCCAGCCAATGCCTTTG
FAS	**F:** GAGGACACTCAAGTGGCTGA<br>**R:** GTGAGGTTGCTGTCGTCTGT
G6Pase	**F:** GACTGTGGGCATCAATCTCC<br>**R:** ACAGGTGACAGGGAACTGCT
GAPDH	**F:** CCATCACCATCTTCCAGGAG<br>**R:** GTGGTTCACACCCATCACAA
GCK	**F:** GGATGCAGAAGGAGATGGAC<br>**R:** GCATCACCCTGAAGTTGGTT
GLUT2	**F:** CAGTGTCTGCTACTGCTCTTCTG<br>**R:** GAGACCTTCTGCTCAGTCGATG
IR	**F:** CCACCAATACGTCATTCACAAC<br>**R:** GGGCAGATGTCACAGAATCAA
PCK1	**F:** CCGTCTGGCTAAGGAGGAA<br>**R:** GGGCATCAAACTTCATCCAG
PCK2	**F:** GGCTGGAAAGTGGAGTGTGT<br>**R:** AGTACACACCGCCATCACTG
PPARγ	**F:** TCAGCTCTGTGGACCTCTCC<br>**R:** ACCCTTGCATCCTTCACAAG

UCP2	**F:** GCATTGGCCTCTACGACTCT<br>**R:** GTCCTGGTATCTCCGACCAC

aP2: adipocyte Protein 2 (fatty acid binding protein); CD36: Cluster of Differentiation 36 (fatty acid translocase); FAS: Fatty Acid Synthase; G6Pase: Glucose 6-phosphatase; GAPDH: Glyceraldehyde 3-phosphate dehydrogenase; GCK: Glucokinase; GLUT2: Glucose transporter type 2; IR: Insulin receptor; PCK1: Phosphoenolpyruvate carboxykinase 1; PCK2: Phosphoenolpyruvate carboxykinase 2; PPARγ: Peroxisome proliferator-activated receptor γ; UCP2: Uncoupling protein 2.
